# Just-in-Time Delivery of Cognitive Behavioral Therapy–Based Exercises: Single-Case Experimental Design With Random Multiple Baselines

**DOI:** 10.2196/69556

**Published:** 2025-07-24

**Authors:** Takeyuki Oba, Keisuke Takano, Daichi Sugawara, Kenta Kimura

**Affiliations:** 1 Human Informatics and Interaction Research Institute, National Institute of Advanced Industrial Science and Technology, 1-1-1 Higashi, Tsukuba, 305-8566, Japan, 81 50-3522-4500; 2 Institute of Human Sciences, University of Tsukuba, Tsukuba, Japan

**Keywords:** ecological momentary assessment, just-in-time adaptive intervention, stress, rumination, cognitive behavior therapy, mindfulness

## Abstract

**Background:**

Just-in-time adaptive interventions (JITAIs) are a promising approach in mental health care given the potential scalability (ie, interventions are offered automatically and remotely) and preciseness (ie, the right interventions are offered at the right moments). Typically, a smartphone app is programmed to assess users’ psychological states in daily life; when a particular state is detected, the app prompts users to engage in specific behaviors. Conceptually, JITAIs hold significant potential for precision health, although there is currently limited evidence in the literature.

**Objective:**

We implemented this scheme as a smartphone intervention for daily stress management, based on cognitive behavioral therapy (CBT), and evaluated its feasibility and efficacy using a single-case experimental design.

**Methods:**

A total of 8 Japanese adults (community sample: 4 women; mean 37.6, SD 13.1 y) were recruited. An AB phase design with multiple random baselines was used, where “A” represents the baseline phase and “B” represents the intervention phase. Throughout the study period (28 d), participants were prompted to indicate their momentary levels of stress (range 0‐100) using a smartphone thrice a day. The baseline phase duration was randomly varied among participants, lasting between 7 and 14 days. The remaining period was used as the intervention phase (14‐21 d), where 6 CBT-based exercises (ie, breath control, mindfulness, relaxation, self-talk, cognitive defusion, and cognitive restructuring) were offered depending on the reported levels of stress.

**Results:**

Approximately 70% (6/8) of the participants perceived the intervention to be useful and helpful. A randomization test detected a statistically significant decrease in reported stress levels after the intervention began (*P*=.005), though this effect was less pronounced when analyzed individually for each participant. Multilevel model analysis detected a significant acute reduction in the momentary level of stress right after completing a CBT-based exercise (pre-exercise: mean 47.98, SD 21.65; post exercise: mean 42.13, SD 19.88; *P*=.03; Cohen *dz*=0.58). Also, a significant reduction in depressive rumination was observed in the postintervention assessment (preintervention: mean 13.00, SD 3.21; post intervention: mean 9.25, SD 2.60; *P*=.01, Cohen *dz*=1.17).

**Conclusions:**

The intervention was feasible and effective in reducing subjective stress (and rumination) in the study sample. The small sample size and the nonclinical nature of the sample may limit the generalizability and implications of the study findings for clinical practice. More evidence should be collected to draw solid conclusions for technical and technological as well as clinical aspects of mobile interventions. Accumulating exemplars with different implementations will clarify how a JITAI can be designed and developed on a mobile platform and how the program can be delivered in the prevention and treatment of mental ill health.

## Introduction

### Background

Digital technologies have become familiar not only in daily life but also in clinical practice for mental health. The electronic and mobile implementation of psychological interventions allows for the remote and ubiquitous delivery of evidence-based care, which is of particular importance to people who have limited access to clinics due to financial or physical constraints [1]. The COVID-19 pandemic has served as a catalyst [2], accelerating the development of digital health care services. These services can be delivered in various formats and platforms, such as computer- or internet-based interventions, via chat systems or smartphone apps, with or without human therapeutic guidance. Stakeholders appear to see mobile implementations as one of the most promising directions, given the number of apps available in the market and the huge amount of investment in recent years [3].

An increasing number of efficacy and effectiveness trials have been published in recent decades, and the results have been synthesized from different aspects. For example, a meta-analysis of digital interventions in general showed a moderate effect on improving depression [4]. Another meta-analysis, with a particular focus on app-based mobile health interventions [5], found small effects on anxiety and depression. A meta-analysis of app-based interventions for moderate to severe depression reported a larger, moderate-sized effect [6]. Overall, the evidence has been mixed, which may be attributed to heterogeneity in the intervention designs and how each intervention (or technique) is delivered.

The rapid development of mobile technologies has expanded the degrees of freedom in implementing care and interventions offered digitally and automatically. Ecological momentary interventions (EMIs) are a general scheme for delivering psychological interventions in daily life, typically for which an app is programmed to obtain information from users and prompt them to engage in specific behaviors at opportunistic times [7-9]. Many EMIs are designed to be just-in-time adaptive interventions (JITAIs), which deliver interventions remotely that are personally tailored and implemented on the basis of an individual’s context (eg, with the GPS in a mobile device), psychophysiology, or responses to questions. As such, a JITAI provides the right treatment (ie, “adaptive”) at the right moment (ie, “just-in-time”) [10]. For the management of mental health, a JITAI typically delivers cognitive behavioral and other specific messages (eg, for behavior activation and cognitive reappraisal) tailored to an individual’s present context and state [11]. The just-in-time feature is of particular importance for patients to become aware of the context in which a maladaptive psychological process (eg, depressive rumination) is triggered and the consequences that maintain it. Appropriate, timely guidance would help them perform functional analyses of their maladaptive behavior and more accurately implement alternative coping [12]. The concept and theoretical basis are not at all novel and have been implemented in existing, in-person psychological treatments. However, the most critical limitation of the traditional approaches is that interventions are offered in brief and infrequent windows of time (eg, in a session at a clinic), which assumes patients’ memory and effort to apply the learned techniques in daily life. Thereby, the JITAI scheme is expected to overcome the therapy-real world gap while delivering relevant support at the time, place, and frequency needed to intervene in symptoms as they arise [13].

Decision rules, which define what interventions to deliver and when [10], constitute the core of EMI. A variety of interventions are offered in EMIs, such as cognitive behavioral therapy (CBT), acceptance and commitment therapy, mindfulness, behavioral activation, and relaxation (for a review, see [14]). Furthermore, different trials have used different logic to define delivery interventions. For example, a JITAI trial assessed participants’ experiences of stressful events and related ruminative thinking several times a day, which were then used to determine which messages and CBT-based support are needed to be delivered [15]. Another JITAI system referred to contextual information (eg, the number of emails and meetings of the day) as well as passive sensing of facial expressions as tailoring variables to determine stress levels [16].

### Objectives

However, EMI and JITAI are still in their infancy, and to the best of our knowledge, there are no established designs (particularly decision rules) that promise the success of an intervention. In other words, an evidence gap exists in the way to determine when and what interventions should be delivered. Interventions were offered, for example, at fixed or semirandom moments [17,18], when stressful events were reported [15], or when environmental and contextual information indicated a high workload of users [16]. Also, the degree of optimization varies across trials—the same interventions (or messages) can be delivered across different occasions, or different interventions can be scheduled according to the values of tailoring variables. Given that no standard or consensus has been established so far, it is pivotal to accumulate exemplars with different implementations and test various decision rules, as there are a number of parameters to optimize when designing a JITAI. The key parameters include the following questions:

1. What interventions or techniques should the system offer in its pool?
2. What psychological and behavioral states should be monitored, and how?
3. What conditions should be implemented to trigger or inhibit an intervention?

This study was designed as a pilot example of JITAI, implementing decision rules to offer different CBT techniques for stress management, depending on the momentary states of app users.

Specifically, we aimed to evaluate the feasibility and efficacy of an app-based intervention with just-in-time prompts using CBT-based exercises for stress reduction. The key idea was to provide an opportunity to engage in CBT exercises (including relaxation, body scans, self-compassion, distancing, and cognitive restructuring) when needed, that is, when elevated stress levels are detected. These CBT techniques (each had an evidence base with or without mobile implementations [13,19]) were offered in the form of stand-alone, self-guided exercises, which could be completed in a few minutes without any support from therapists or other health care professionals. This quick-to-go format is crucial for JITAI, similar to homework in traditional CBT [20], as participants are prompted to engage with it in daily life. Furthermore, our pilot app was designed to offer different exercises according to the detected levels of stress. This tailoring was important because stress has intraindividual, temporal dynamics, and appropriate coping strategies may differ across the levels and types of stress [21,22]. It is not always possible for individuals to make a rational decision on how to cope with their stress. A survey on general practitioners showed that even health care professionals use ineffective coping strategies (eg, avoidance strategies and self-blame) under highly stressful situations [23], which highlights the need for tailored, context-specific guidance.

To this end, we conducted this pilot, proof-of-concept trial to test the feasibility (eg, adherence, acceptability, and usability) of the JITAI approach as well as its efficacy in reducing stress over a 2- to 3-week intervention. A single-case experimental (AB phase) design was used with random multiple baselines (baseline for 7‐14 d vs intervention for 14‐21 d). For the feasibility, we were particularly interested in how participants would feel about the timing and frequency of the prompts, which may affect the perception of usefulness and willingness to continue the app use. One of the known challenges for mental health apps is the low retention rate [24]—this issue could be resolved by the regular prompting of JITAIs (eg, to maintain users’ interests), but too much prompting or inappropriate delivery timing may have adverse effects leading to dropout. For efficacy, we hypothesized that participants would show significant decreases in subjective stress over time.

## Methods

### Participants

Eight adults (mean age 37.6, SD 13.1 y; 4 women) were recruited from inhabitants of Ibaraki Prefecture and its environs (middle-north regions in Japan) early in 2024. The study was advertised online (eg, via portal sites and emails), and those interested were invited to an online eligibility assessment. Participants were assessed for their current levels of subjective stress by responding to an item from the Perceived Stress Scale (PSS; *"*In the last week, how often have you felt nervous and stressed?*"*) [25] by indicating the most applicable of the 5 response options: 0=never, 1=almost never, 2=sometimes, 3=fairly often, and 4=very often. Those who scored 3 or 4 on the PSS, indicating they felt nervous and stressed fairly often or very often, were eligible to participate. Other inclusion criteria were as follows: (1) aged 20‐64 years, (2) expecting no major life changes right before or during the study participation, (3) not pregnant or lactating, (4) being in good health with no history of dysautonomia, depression, mania, bipolar disorder, or facial paralysis, (5) no medication with tranquilizers or antihypertensive agents, (6) not under hormone replacement therapy, (7) no oral contraceptives, and (8) no habit of physical activity and exercise (this criterion was used for an overarching study). Recruitment continued until the planned sample size was achieved. The sample size was determined pragmatically (considering the resources of the recruiting institution) due to the pilot nature of this study. However, a simulation study [26] examining the power of randomization tests in multiple baseline designs suggested that a sample size (N) of 6‐10 participants would be sufficient to detect an effect of Cohen *d*=0.60‐1.0 with a sufficient power (0.80).

### Study Design

**Figure 1.**
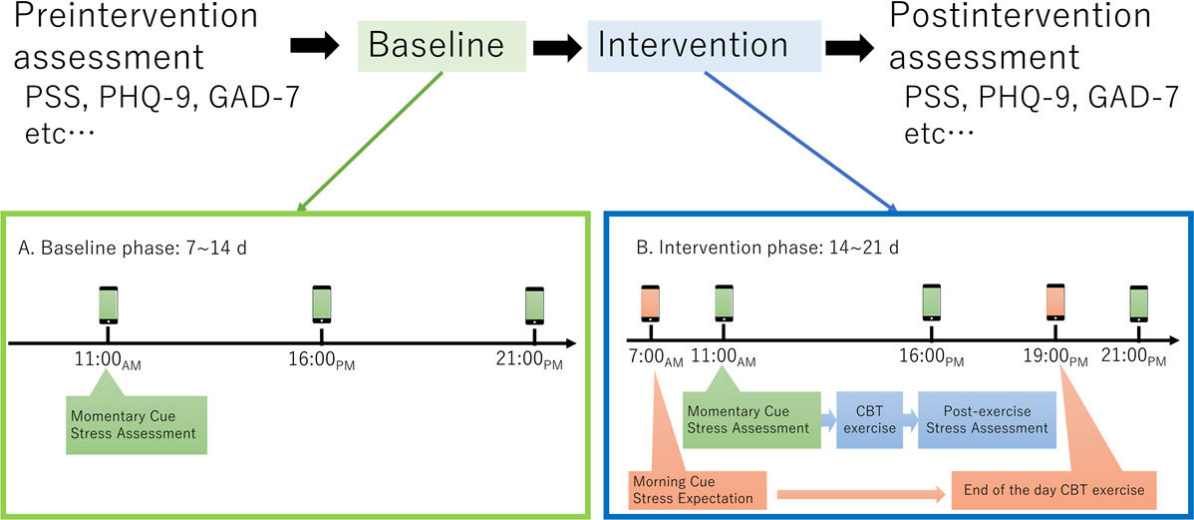
Schematic flow diagram of the study. An AB design was used (28 d in total for each participant) with the baseline length randomized across participants (7‐14 d). CBT: cognitive behavioral therapy; GAD-7: Generalized Anxiety Disorder-7 Scale; PHQ-9: Patient Health Questionnaire-9; PSS: Perceived Stress Scale.

An “AB” design with random baselines was used (Figure 1). The entire study period was 28 days (4 wks) for each participant. The baseline phase duration was randomly varied among participants, lasting between 7 and 14 days. The remaining period was used as the intervention phase (14‐21 d). Participants were not informed when the intervention would be started (single blinding). Throughout the 28-day study period, participants received 3 signals each day at fixed times (11 AM, 4 PM, and 9 PM) on their smartphones with an EMA app, m-Path [27], being installed. These momentary cues prompted participants to indicate their current levels of subjective stress using a 0‐100 slider (0=not at all, 100=very much). The exact prompt states the following:

*We would like to ask you about your current stress level. Please rate your current stress using the slider below*.

During the intervention phase, participants received in-app guidance for CBT exercises (see the next section “CBT Exercises”) depending on the reported momentary levels of subjective stress. For example, if participants scored 80 or above on the stress slider, they received a recommendation for muscle relaxation, guided by a tutorial video (3‐9 min long each). Participants were allowed to engage in an exercise that was not recommended at this moment (eg, performing breath control instead of or in addition to muscle relaxation) or skip the opportunity if they were too busy. In addition, if participants indicated low levels of stress (ie, <40 on the slider), they received no specific recommendation for exercise.

At the end of the exercises, participants were asked to specify the exercises in which they were actually engaged (this prompt appeared even when no recommendation was presented). Specifically, they indicated any applicability from the list of exercises (ie, breath control, mindfulness, progressive muscle relaxation, compassionate self-talk, cognitive defusion, and cognitive restructuring) with *"*other and skipped (too busy)" response options. Subsequently, participants rated their current stress levels again using a 0‐100 slider. Postexercise stress levels were subjected to randomization tests as the primary outcome.

In addition to the momentary cues, participants were prompted each morning to estimate how busy they would be today; depending on the estimated levels, they received another in-app guidance for CBT exercise at 7 PM, on the day. The morning cue stated:

*How busy do you expect to be today? Use the slider below to indicate how busy you expect to be today*.

We asked how busy they would be instead of how stressful the day would be because we were not sure how accurately participants could estimate the stress level of the day; instead, estimating the extent of busyness was deemed more reliable and easier for participants, as they could refer to, for example, their schedules.

### CBT Exercises

Different CBT exercises were prompted depending on the reported levels of stress in response to the momentary and morning cues. Table 1 summarizes the individual exercises and decision rules. Among the techniques used in CBT and related programs [28,29], we implemented the following 6 exercises into the current program: breath control [30], mindfulness [31], progressive muscle relaxation [32], compassionate self-talk [33], leaves on a stream exercises [34], and cognitive restructuring [29]. These techniques are suitable for mobile implementation as stand-alone exercises, each instructed via videos lasting between 3.5 and 9.0 minutes.

**Table 1.** Decision rules defining exercises prompted for different levels of stress.

Cue, stress, or busyness level threshold	Exercise	Description, example instruction
Momentary cues^a^		
Stress level		
0‐39	No exercise prompted	No exercise prompted
40‐59	Breath control	Take deep breaths while counting the numbers in your mind
60‐79	Mindfulness (focusing on breath)	Pay attention to your breathing and savor the sensation
80‐100	Progressive muscle relaxation	Reduce stress and anxiety by progressively tensing and then relaxing different muscle groups in the body
Morning cues^b^		
Busyness level		
0‐39	No exercise prompted	No exercise prompted
40‐59	Compassionate self-talk	Repeat in your mind a phrase that is warm and compassionate toward self
60‐79	Leaves on a stream (cognitive defusion)	Imagine leaves flowing down a river and observe the thoughts and feelings that arise
80‐100	Cognitive restructuring	Recall negative events, write down thoughts and feelings, and assess whether your evaluations are accurate

a Participants responded to momentary cues thrice a day, and exercise was offered right after each momentary cue.

b Participants responded to a morning cue each day (7 AM) and indicated how busy they would be today; exercise was offered at 7 PM each day.

The first 3 exercises (breath control, mindfulness, and muscle relaxation) were prompted by momentary cues. These quick-to-go exercises (less cognitive, typically with a particular focus on the body and meditation) were selected because momentary cues were scheduled thrice a day and each exercise should not be too demanding. Another focus was on the levels of stress, and we programmed breath control for low levels of stress, mindfulness for moderate levels, and muscle relaxation for high levels. We expected that the participants (a community sample) would experience low levels of stress most frequently, for which a hands-free, light-touch exercise was deemed appropriate. Therefore, the breath-control tutorial video was the shortest (3.8 min) of the three (9.0 and 5.5 min for mindfulness and progressive muscle relaxation, respectively).

The other three exercises (compassionate self-talk, leaves on a stream exercises, and cognitive restructuring) were programmed for morning cues and were to be completed in the evening (at 7 PM or later). These imaginative or cognitive exercises were performed once a day (which is less frequent than those for the momentary cues) and thus were meant to be more cognitively effortful and typically required a longer time to complete. Studies suggest that the successful use of cognitive coping, particularly reappraisal, may produce significant cognitive costs, and people choose reappraisal less frequently than other strategies (eg, suppression and distraction) to regulate their emotions [35,36]. Therefore, we programmed cognitive exercises to be offered in the evening (deemed to be a quieter moment with fewer distractors than midday), not right after experiencing stress (ie, momentary cues). Compassionate self-talk was prompted when participants indicated that they would be somewhat busy today in response to a morning cue. Leaves on a stream, exercises, and cognitive restructuring were recommended when participants expected that the day would be tougher. Also, cognitive restructuring requires individuals to recall a specific negative event, and thus, this exercise was regarded as being suited to the busiest day when they could easily spot a stressor to target. These decision rules were determined somewhat arbitrarily, but compassionate self-talk was assumed to be less effortful and a better fit on the day when light stress was foreseen compared to the other two.

### Measures

We administered two types of assessments: momentary and pre- and postintervention assessments. Our primary outcome was the momentary postexercise levels of subjective stress reported following the momentary cues, which were sent three times daily over the 28-day study period. The pre- and postintervention assessments included questionnaires on subjective stress (over the past month), psychopathological symptoms and traits, and well-being. The postintervention assessment also included feasibility measures to clarify how acceptable and appropriate the intervention was for each participant. Note that we did not examine the internal consistency of each measure, given the small sample size. However, all the measures used here have been shown to have good psychometric properties.

#### The PSS

Subjective stress levels were assessed using the PSS, which consists of 10 items [25]. The scale asks how respondents felt and thought during the past month (eg, "how often have you been upset because of something that happened unexpectedly?"). Each item is rated on a 5-point scale (0=never and 4=very often), and the total score is calculated. A validation study documented a mean score of 20.3 (SD 5.4) in a Japanese sample [37], in which a PSS score > mean 31, SD 2) can be interpreted as a high level of subjective stress.

#### Brief Patient Health Questionnaire

Depressive symptoms were assessed using the brief Patient Health Questionnaire-9 (PHQ-9) depression module [38]. This scale consists of 9 items representing depressive symptoms rated using a 4-point scale (0=not at all and 3=nearly every day). The sum score was calculated and interpreted with cutoff scores of 5, 10, 15, and 20, indicating mild, moderate, moderately severe, and severe depressive symptoms, respectively.

#### Seven-Item Anxiety Scale

Anxiety and symptoms of generalized anxiety disorder were assessed using the Generalized Anxiety Disorder-7 Scale (GAD-7) [39]. Each item represents typical generalized anxiety disorder symptoms (eg, nervousness and excessive worry), rated using a 4-point scale (0=not at all and 3=nearly every day). The sum score was calculated and interpreted with cutoff scores of 5, 10, and 15, indicating mild, moderate, and severe levels of anxiety, respectively.

#### Response Styles Questionnaire

Ruminative response styles to depressed mood were assessed using the Response Style Questionnaire (RSQ) [40,41]. The brooding subscale was the target of our interest, which is known to be predictive of depression and anxiety (eg, [42]). This subscale has 5 items (eg, Think “Why can’t I handle things better?”), and each is rated using a 4-point scale (1=almost never and 4=almost always). The sum score was calculated.

#### Hedonic and Eudaimonic Motives for Activities Scale

Hedonic and eudaimonic conceptions of well-being were assessed using the Hedonic and Eudaimonic Motives for Activities (HEMA) scale [43]. The HEMA scale has 2 subscales: hedonic and eudaimonic. The former assesses pleasure and the absence of pain and is subdivided into 2 factors: seeking relaxation (2 items) and seeking pleasure (3 items). The eudaimonic subscale consists of 4 items, reflecting, for example, authenticity and excellence. Each item is rated using a 7-point scale (1=not at all and 7=very much), and the sum scores are calculated for the eudaimonic, seeking-relaxation, and seeking-pleasure subscales.

### Feasibility

Immediately after the intervention phase, participants rated the feasibility of the intervention online. Specifically, they rated the following aspects using a 7-point scale (1=not at all and 7=very much): the intervention was (a) satisfactory, (b) useful, (c) helpful, (d) suited to me, (e) appropriate timing, (f) practical, and (g) I would like to continue using this intervention system [44].

### Procedure

Eligible participants were individually invited to the laboratory for a briefing session. They received study instructions and mobile devices (a smartphone and activity tracker) for assessment and intervention. After providing written informed consent, participants completed questionnaires on stress and symptomatology (eg, the PSS and PHQ-9). The baseline phase began on the day after the briefing. The randomization sequence for the baseline length was generated prior to recruitment and masked to the participants. Upon completion of the intervention phase, participants completed the postintervention questionnaires (online) and returned their laboratory devices in exchange for compensation for study participation.

### Statistical Analyses

Momentary levels of stress, reported 3 times daily (for the intervention phase, those reported immediately after the exercises), were aggregated within each day to reduce the proportion of missing values when passing the data to the randomization tests. The aggregated scores were then subjected to randomization tests to establish the effect of the intervention, which was operationalized as the mean difference in the stress score between the baseline and intervention phases. A randomization test permutes the condition assignments of each assessment occasion for a person, which mimics the random assignment used to test a null hypothesis about the treatment effect in a randomized experiment [45]. For example, for a participant who received an assignment sequence of AABBBB for T1-T6 assessment occasions (ie, the first 2 occasions were the baseline and the following 4 were in the intervention phase), the observed test statistic is given by the mean of T1-T2 minus that of T3-T6. Then, a randomization test calculates the test statistics for each possible permuted sequence (eg, ABBBBB, AAABB, and AAAAB) to generate a randomization distribution, on which the observed test statistic (here: T1-T2 minus T4-T6) is located to test the null hypothesis, for example, no treatment effect exists. The *P* value is given as the proportion of the observed value equal to or smaller than the values from the permutated sequences. We set the α level to .05 and generated random permuted sequences 5000 times. Randomization tests were conducted on each participant and group of participants [46]. The R (R Core Team) package Single-Case Randomization Tests (SCRT) [45] was used to conduct the randomization tests.

As an exploratory analysis (not preregistered), a multilevel model was estimated to test whether the momentary level of stress was acutely decreased right after each CBT exercise. The model specified stress as the outcome and time (pre- vs postexercise) as the predictor with the random intercept to allow the intercept to vary across participants. Similarly, we tested whether the stress level during the baseline phase would differ from the pre-exercise level during the intervention phase. Multilevel modeling was conducted using “lmer4” [47] and “lmerTest” packages [48].

We did not specify in the preregistered protocol how missing values would be handled, as we did not have a clear prospect for how good the compliance would be among the studied population with this specific study design. After analyzing the data, we found that several participants had missing responses for more than half of the momentary cues (see the section “Dropout and Dose”). Therefore, we concluded that imputations would not be helpful at the within-person, momentary level of the data. Also, the randomization tests were meant for the efficacy but not for the effectiveness of our pilot intervention, and therefore, a per-protocol analysis was regarded to be more appropriate than an intention-to-treat approach. To this end, the randomization tests were performed on the day-level data, with participants’ momentary responses being aggregated for each day. The exploratory, multilevel modeling analysis was conducted with the maximum likelihood estimation while leaving out a participant who showed extremely low compliance (eg, dropout and dose). Although this estimation approach is robust under the assumption of missing at random, we would not argue that our results were unbiased as we did not have sufficient contextual information to gauge the randomness or underlying mechanisms of missingness; like other EMIs, it was highly likely that participants responded to momentary (and morning) cues only when they were available for that.

### Ethical Considerations

The study protocol was approved by the Ethics Committee of the National Institute of Advanced Industrial Science and Technology (ID 2022‐1240). The registered protocol is available from Open Science Framework [49], and we adhered to the SCRIBE (Single-Case Reporting Guideline In Behavioural Interventions) checklist [50] (see Checklist 1). Each participant received detailed study information about the procedure, aim, and their rights in the briefing session. They were also informed that they had the option to withdraw from study participation without any consequences at any point in the study. Afterward, they provided written informed consent prior to completing the baseline questionnaires. The privacy and confidentiality of participants were ensured by pseudoanonymizing all collected data. Each participant was recognized by a unique participant ID in the collected data, and personal information (eg, participants’ names and emails) was saved in a separate file. A reference list was made digitally to link the ID and personal information, which was accessible only by the first author and was deleted at the end of the data collection. Participants received JPY 15,000‐20,000 (US $100‐$140) as compensation at the end of the study.

## Results

### Demographics and Baseline Characteristics

Eight participants who met the inclusion criteria were enrolled, five of whom were married and had a child or children. Four participants were currently unemployed. All participants had received higher education, namely, graduated from a 2-year college, university, or above. Regarding baseline levels of stress and psychopathology, all participants had experienced high levels of stress, four had mild levels of depressive and anxiety symptoms, and one had moderate levels of symptoms (Table 2).

**Table 2.** Preintervention characteristics of each participant.

ID	Baseline length (day)	Age (years)	Gender^a^	PSS^b^	PHQ-9^c^	GAD-7^d^	RSQ^e^	HEMA scale^f^ Eud^g^	HEMA scale Ple^h^	HEMA scale Relx^i^
1	7	52	Men	50	7	9	12	21	18	18
2	8	43	Men	42	4	1	17	22	18	16
3	9	22	Men	54	3	7	14	28	20	18
4	10	23	Men	46	9	8	17	19	16	22
5	11	23	Women	58	7	4	13	19	18	21
6	12	52	Women	31	4	2	7	21	18	24
7	13	39	Women	53	13	12	12	22	18	26
8	14	47	Women	37	4	6	12	17	17	13
Mean (SD)	—^j^	37.6 (13.1)	—	46.4 (9.2)	6.4 (3.4)	6.1 (3.7)	13.0 (3.2)	21.1 (3.3)	17.9 (1.1)	19.8 (4.3)

a Gender: self-identified.

b PSS: Perceived Stress Scale.

c PHQ-9: Patient Health Questionnaire-9.

d GAD-7: Generalized Anxiety Disorder-7 Scale.

e RSQ: Response styles questionnaire, brooding scale.

f HEMA: Hedonic and Eudaimonic Motives for Activities.

g Eud: eudaimonia.

h Ple: pleasure.

i Relx: relaxation.

j Not applicable.

### Dropout and Dose

All participants completed the pre- and postintervention assessments (no dropouts were observed). No adverse events were reported. One participant (ID 4) showed low compliance (responded to 9/30, 30% momentary cues during the baseline phase and 2/72, 3% momentary cues during the intervention phase). While there is no established standard for the minimum number of observations needed for a randomization test as far as we know, 3‐5 observations per phase may be a practical minimum [51]. The test on ID4, which had only 2 observations in the intervention phase, was thus deemed unreliable, and their data were excluded from statistical analyses. The remaining 7 participants responded to 75.6% (range 44.4%‐90.5%) of the momentary cues in the baseline phase and 59.7% (range 28.1%‐79.4%) in the intervention phase. They showed lower compliance with morning cues: 44.3% (range 0%‐92.9%). The frequency of recommendations and number of engagements for each type of exercise are shown in Table S1 in Multimedia Appendix 1. Breath control and mindfulness were recommended and performed most frequently. In other words, participants hardly experienced the highest (80 or above) levels of stress during the intervention period. Overall, 4 participants performed breath control more frequently than recommended. Similarly, 3 participants practiced muscle relaxation spontaneously even when not explicitly recommended.

### Feasibility

Each aspect of feasibility was rated using a 7-point scale (1=not at all to 7=very much), and we counted the number of participants who indicated 4 or higher as an indication of endorsement (Table S2 in Multimedia Appendix 1). Most participants perceived the current intervention as satisfactory (63%), useful (75%), helpful (75%), and practical (63%). In addition, most participants reported that they would like to continue the intervention even after the study (75%). However, 7 out of 8 (88%) participants disagreed that the momentary and morning cues were delivered at appropriate times, suggesting that timing remains a challenge.

### Intervention Effect

**Figure 2.**
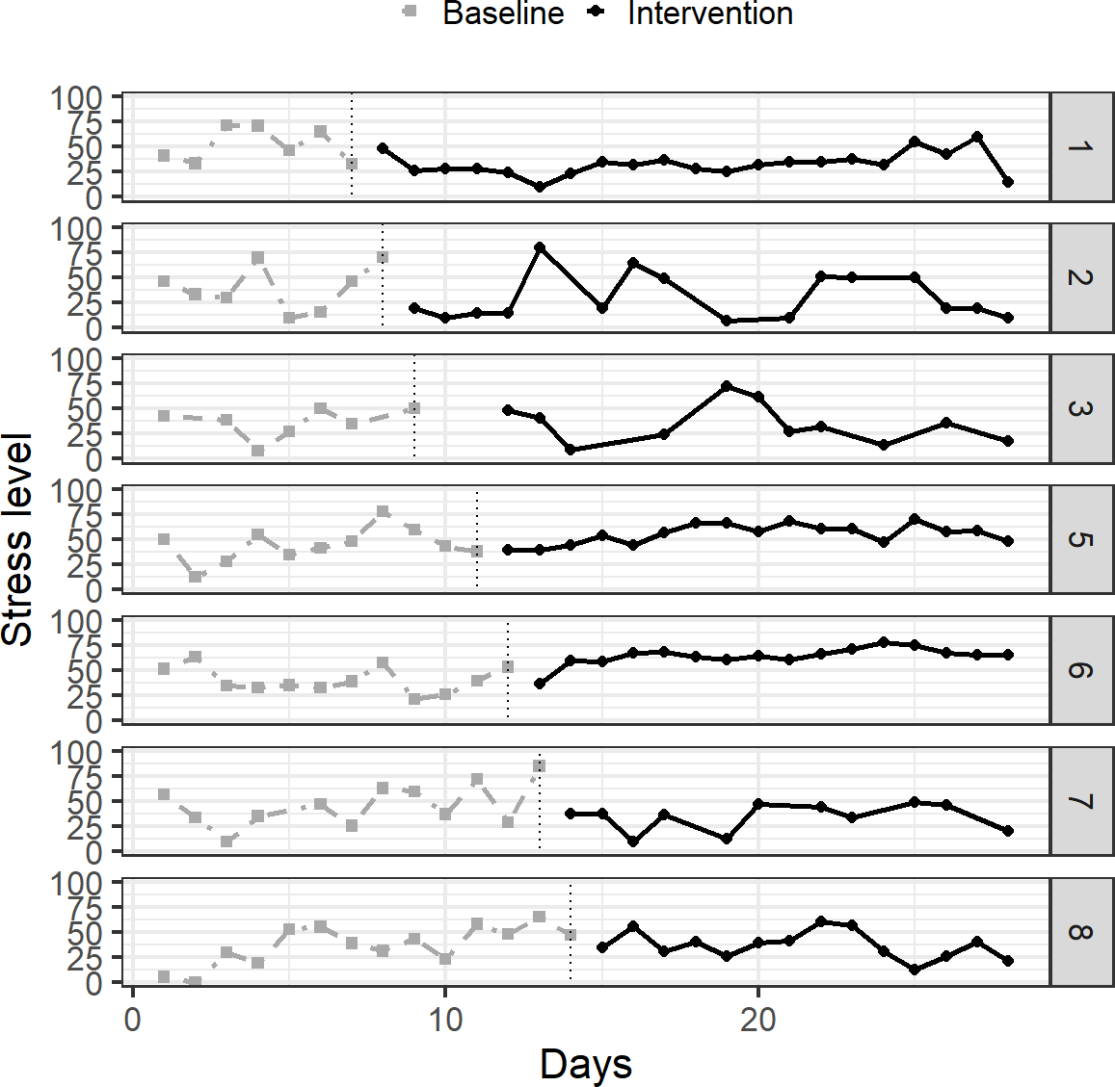
Changes in stress level as a function of time.

Figure 2 illustrates how the stress levels (for the intervention phase, stress reported immediately after each exercise) changed over time. Participants did not show a clear, acute change in the reported stress levels; however, 3 participants (ID 1, 2, and 7) appeared to have experienced reductions, whereas 2 participants (ID 5 and 6) showed slight increases during the intervention phase.

The randomization test across all participants (Table 3) showed a significant decrease in subjective stress from the baseline to the intervention phase (*P*=.005), rejecting the null hypothesis of no change. Note that *P* values were calculated for the test statistics, M (intervention)–M (baseline), with the null hypothesis, M (intervention)=M (baseline); and that ID4 was not analyzed because of low compliance. However, randomization tests for individual participants did not show significant reductions (all *P*>.05), consistent with the visual inspection of Figure 2, where no clear changes or inflection points were observed. In Figure 2, each panel indicates the mean stress levels per day for each participant (ID 1‐8, except for ID 4, which was excluded due to low compliance).

**Table 3.** Means (SDs) of stress levels reported by each participant and the results of randomization tests.

ID	Baseline phase, mean (SD)	Intervention phase, mean (SD)	*P* value
1	51.8 (17.2)	33.0 (11.8)	.25
2	40.3 (22.5)	30.8 (23.0)	.13
3	35.9 (14.8)	34.8 (19.8)	.63
4	—^a^	—	—
5	44.5 (17.1)	55.4 (9.90)	.52
6	40.6 (13.1)	64.2 (9.06)	.63
7	46.1 (21.7)	34.0 (13.8)	.13
8	37.0 (19.8)	36.8 (13.9)	.12
All participants	42.3 (5.6)	41.3 (13.0)	.01

a Not applicable.

As an exploratory analysis, we tested whether the stress level was acutely decreased from the pre- to postexercise moments. A multilevel model analysis detected a significant decrease, fixed effect=−5.77; *t*_4.57_=3.038; *P*=.03; Cohen *dz*=0.582 (pre-exercise: mean 47.98, SD 21.65; post exercise: mean 42.13, SD 19.88). Another multilevel model revealed no significant change from the baseline phase to the pre-exercise moments during the intervention phase (fixed effect=1.62; *t*_6.06_=0.315; *P*=.76; Cohen *dz*=0.013; baseline: mean 44.34, SD 20.46).

We calculated the means and SDs for each measure administered at the pre- and postintervention assessments (Table S3 in Multimedia Appendix 1). Neither well-being nor psychopathology symptoms showed notable changes (Cohen *dz*<0.35; *P*>.36), except for rumination, which showed a statistically significant reduction over time (Cohen *dz*=1.17; *P*=.01). Although this result should be interpreted carefully, given the small sample size, the current intervention may help control depressive cognitive styles.

## Discussion

### Feasibility and Adherence

This pilot trial aimed to establish the feasibility and efficacy of the JITAI in reducing subjective stress among 8 Japanese adults experiencing high levels of subjective stress at enrollment. Participants perceived the intervention as satisfactory and helpful; overall, it is appropriate to conclude that the intervention was acceptable. Breath control and mindfulness were the most frequently recommended exercises by the system (because participants typically indicated that they were experiencing low-to-moderate levels of stress in response to momentary cues) and thus were the most frequently performed by participants. Several participants spontaneously performed breath control and relaxation, even when these exercises were not explicitly recommended. These findings are in line with the feasibility and acceptability reported in the literature for mobile (particularly mindfulness) interventions. A JITAI study for smoking cessation [52] delivered brief mindfulness strategies for stress management via smartphone, and participants reported that those strategies were helpful and they were likely to continue to use the strategies. Another trial that offered brief body scans via smartphone documented excellent adherence and continued interest of participants in mindfulness practice [53]—more than half of the participants spontaneously maintained mindfulness practice even after the active intervention phase. Good retention and engagement have also been reported in a JITAI intervention targeting repetitive negative thinking [13].

However, the feasibility evaluation revealed a practical issue: participants found that the cues were not sent at the appropriate time. There is an ongoing debate on how to optimize and personalize the intervention timing for an electronic and mobile health care system. In general, it is thought that delivering interventions at the right moments (eg, considering participants’ schedules, circadian rhythms, and lifestyles) would increase the likelihood of accepting and adopting the intervention’s recommendations, thereby increasing the adherence rate [54,55]. In this study, one participant failed to respond to most (around 90%) of the momentary cues and therefore missed almost all the opportunities to perform the CBT-based exercises. The remaining 7 participants showed good compliance in responding to momentary (sent at 11 AM, 4 PM, and 9 PM each day) and morning cues (at 7 AM), even though they perceived that these were not the best moments to do so. Participants may have been intrinsically motivated to engage in CBT-based exercises whenever the cues were sent; however, it should be noted that all participants were paid volunteers.

### Efficacy

The overall effect of the intervention on momentary subjective stress levels merged across all participants was statistically significant. This result implies that the intervention is effective in reducing subjective stress. However, the randomization tests on individual participants failed to detect significant effects, which may suggest that the size of the effect was too small to be detected with the current design, the statistical power was not sufficient with the number of observations per participant, or both. This study used across-participants random multiple baselines, which requires at least two participants to randomize by nature. As such, the test on multiple participants fits the study design better than the individual-participant tests, which do not consider the manipulation (ie, randomization) in the baseline length. Importantly, it is known that both the larger number of participants and the wider range of possible start moments of intervention (=possible lengths of baseline) lead to a higher power [26]. Another possibility is that the effect of the exercises was short-lived. Indeed, we found that there was a statistically significant decrease in stress right after completing a CBT exercise, but this decrease did not appear to stay for the entire intervention period.

It is also noteworthy that 2 participants (ID 5 and 6) experienced increases (but did not reach statistical significance) in the intervention phase. We do not have data that readily explain these unexpected increases; both participants received a fair dose, which was comparable to that of other participants (ID 5: breath control 17 times, ID 6: mindfulness, and cognitive defusion 11 times). It is too early to conclude that these exercises had adverse effects on the participants, but the data suggest that they were not responsive to these CBT techniques. In general, single-case experimental design, combined with repeated measurements, helps better understand how an outcome (here: stress) changes within an individual over an intervention course, compared with a typical experimental (eg, parallel group) design that averages participants' behaviors as a group [56]. Such contextualization may be the first step to explore how the outcome change is manifested in daily life and to explore who is responsive to what interventions. Establishing the optimal prescription of CBT techniques was beyond our scope, but integrating the P4 health spectrum into a JITAI system is crucial to address the variability in how individuals respond to psychological interventions [57].

Another interesting finding was that participants experienced significant reductions in depressive rumination. This effect was assessed as the difference between the two (pre- vs postintervention) time points, and thus, should be interpreted carefully, given the low statistical power. That said, it is fair to say that the current intervention might include a factor that helps reduce cognitive vulnerability (ie, rumination), such as mindfulness. Mindfulness interventions, even in a brief format or embedded in an EMI, are known to be effective not only in improving symptoms but also in reducing depressive rumination and repetitive negative thinking [53,58]. Given that rumination is understood as a mental habit sustained by learned context-response associations (eg, rumination triggered when being alone), JITAI may be a good platform to identify cuing contexts in daily life and to replace an unhelpful ruminative response to the cues with a more helpful response [59]. The just-in-time nature would well suit this scheme (ie, counterconditioning to establish a new context-response association), which allows patients to perform repeated practice at using an alternative incompatible coping strategy immediately after a triggering contextual cue is detected. However, our pilot system was not designed to collect contextual and environmental information; also, we cannot conclude which factors (mindfulness, cognitive restructuring, relaxation, etc) were the active ingredients to facilitate the learning of new compelling behaviors. Future research should explore the contextual cues to target and the (combinations of) techniques to offer for further optimization (eg, using a fractional factorial design [60]).

### Limitations

This study had some limitations. First, the nonclinical nature of the sample may have limited the clinical implications of the study findings. The mobile implementation of CBT techniques, especially with a just-in-time approach, can guide daily stress management for nonclinical or at-risk individuals. However, it remains necessary to examine whether this approach works in clinical settings as a stand-alone self-help intervention or as an add-on to therapist-guided treatment. Moreover, since our sample consisted only of Japanese adults, this may limit the generalizability and replicability of our findings across different populations. Future research should target a wider range of users, such as children, adolescents, older adults, and those with different cultural backgrounds. This may involve system-wise modifications (eg, digital literacy and technology acceptance [61]) and content-wise adaptations of the implemented CBT techniques (eg, used language, acronyms, and vignettes; see for a meta-analysis of cultural adaptations [62]). Second, although the single-case design is a powerful tool for investigating the intervention effect in a small sample, it is appropriate to proceed to a larger-scale trial with appropriate controls and long-term follow-ups to establish the efficacy and effectiveness of the intervention. Third, a sampling bias may have affected the results. Overall, we found good compliance, and it is possible that the participants enjoyed the intervention and were intrinsically motivated to participate. However, all participants were paid volunteers and adherence (or user engagement) may not be readily generalized to real-life settings.

### Conclusions

The current JITAI implementation of CBT techniques was generally accepted by the participants and was effective in reducing subjective stress and depressive rumination. An important direction for future research is to develop a reliable system to automatically detect psychological distress and stress (eg, [63]) that can be embedded in the decision rule (ie, if the sensor detects elevated stress, it triggers a particular intervention). Technological sophistication will improve the usability, adherence, and effectiveness of the intervention.

## Supplementary material

10.2196/69556 Multimedia Appendix 1 Additional tables.

10.2196/69556 Checklist 1 Completed SCRIBE (Single-Case Reporting Guideline In Behavioural Interventions) checklist.

## Abbreviations

CBT: cognitive behavioral therapy
EMI: Ecological momentary intervention
GAD-7: Generalized Anxiety Disorder-7 Scale
HEMA: Hedonic and Eudaimonic Motives for Activities
JITAI: Just-in-time adaptive intervention
PHQ-9: Patient Health Questionnaire-9
PSS: Perceived Stress Scale
RSQ: Response Style Questionnaire
SCRIBE: Single-Case Reporting Guideline In Behavioural Interventions
SCRT: Single-Case Randomization Tests
