# Supplementary material for: Just-in-Time Delivery of Cognitive Behavioral Therapy–Based Exercises: Single-Case Experimental Design With Random Multiple Baselines
Source: JMIR Form Res. 2025 Jul 24;9:e69556. doi: 10.2196/69556 (PMC12288703; doi:10.2196/69556)
Supplement: Multimedia Appendix 1 [file formative-v9-e69556-s001.docx]

**Just-in-time Delivery of Cognitive Behavior Therapy Based Exercises: A Single Case Experimental Design with Random Multiple Baselines**

**Supplementary materials**

Takeyuki Oba^1^*, Keisuke Takano^1^*, Daichi Sugawara^2^, and Kenta Kimura^1^

^1^ Human Informatics and Interaction Research Institute, National Institute of Advanced Industrial Science and Technology (AIST), Japan

^2^ University of Tsukuba, Japan

**Table S1. Frequency of exercises performed (and recommended) for each participant**

| ID | Breath | Mindful | Relaxation | Self-talk | Defusion | Restruct | None |
| --- | --- | --- | --- | --- | --- | --- | --- |
| 1 | 24 (11) | 10 (15) | 1 (1) | 2 (0) | 2 (2) | 0 (0) | 14 (26) |
| 2 | 11 (4) | 3 (3) | 7 (3) | 2 (2) | 3 (3) | 2 (2) | 5 (17) |
| 3 | 8 (5) | 1 (1) | 2 (1) | 0 (0) | 0 (0) | 1 (0) | 5 (9) |
| 4 | 1 (0) | 0 (0) | 0 (0) | 0 (0) | 0 (0) | 0 (0) | 1 (2) |
| 5 | 17 (18) | 7 (11) | 0 (0) | 1 (4) | 0 (0) | 0 (0) | 9 (14) |
| 6 | 3 (3) | 11 (28) | 3 (4) | 0 (0) | 11 (11) | 2 (5) | 21 (3) |
| 7 | 1 (8) | 1 (2) | 2 (1) | 0 (0) | 0 (0) | 0 (0) | 16 (12) |
| 8 | 8 (7) | 7 (10) | 0 (0) | 1 (0) | 6 (6) | 0 (0) | 20 (23) |

Note. The participants were allowed to engage in any exercise on each occasion, regardless of the recommended exercise. Breath control (Breath), Mindfulness (Mindful), and Progressive muscle relaxation (Relaxation) were offered when low, moderate, or high stress levels were reported (momentary cues), respectively. Compassionate self-talk (Self-talk) leaves on a stream (Defusion), and cognitive restructuring (Restruct) were offered when low, moderate, or high levels of busyness were expected (morning cues).

**Table S2. The means (SDs) of the feasibility measures and the number of participants who endorsed each aspect of the feasibility (indicated 4 or higher on the 7-point scale)**

| Variable | Mean (SD) | Endorsement N(%) |
| --- | --- | --- |
| Satisfactory | 4.6 (1.6) | 5 (62.5) |
| Useful | 4.8 (1.6) | 6 (75) |
| Helpful | 4.9 (1.4) | 6 (75) |
| Suited | 4.4 (1.8) | 4 (50) |
| Timing | 2.9 (1.2) | 1 (12.5) |
| Practical | 4.8 (2.3) | 5 (62.5) |
| Willing to continue | 5.0 (1.8) | 6 (75) |

**Table S3. Pre- to post-intervention changes (N = 8)**

| Variable | Pre-intervention | Post-intervention | *t* | *p* | *Cohen’s dz* |
| --- | --- | --- | --- | --- | --- |
| PSS | 46.38 (9.21) | 43.38 (8.43) | 0.760 | 0.472 | 0.269 |
| PHQ-9 | 6.38 (3.38) | 5.50 (3.38) | 0.813 | 0.443 | 0.287 |
| GAD-7 | 6.13 (3.68) | 5.88 (4.19) | 0.306 | 0.769 | 0.108 |
| RSQ (brooding) | 13.00 (3.21) | 9.25 (2.60) | 3.319 | 0.013 | 1.173 |
| HEMA |  |  |  |  |  |
| Eudaimonia | 21.13 (3.27) | 20.00 (5.40) | 0.672 | 0.523 | 0.238 |
| Pleasure | 17.88 (1.13) | 16.63 (3.46) | 0.978 | 0.361 | 0.346 |
| Relax | 19.75 (4.30) | 19.63 (5.07) | 0.054 | 0.958 | 0.019 |

Note. PSS = Perceived Stress Scale; PHQ-9 = Brief Patient Health Questionnaire; GAD-7 = 7-item Anxiety Scale; RSQ = Response Styles Questionnaire, Brooding Scale; HEMA Eud, Ple, Relax = Hedonic and Eudaimonic Motives for Activities scale: Eudaimonic, Seeking-pleasure, Seeking-relaxation subscales, respectively.
