# Supplementary material for: Just-in-Time Delivery of Cognitive Behavioral Therapy–Based Exercises: Single-Case Experimental Design With Random Multiple Baselines
Source: JMIR Form Res. 2025 Jul 24;9:e69556. doi: 10.2196/69556 (PMC12288703; doi:10.2196/69556)
Supplement: Checklist 1 [file formative-v9-e69556-s002.docx]

| Topic | Description | Note (relevant page number and section) |
| --- | --- | --- |
| TITLE and ABSTRACT | | |
| 1 Title | Identify the research as a single-case experimental design in the title | P.1, “…A Single Case Experimental Design with Random Multiple Baselines” |
| 2 Abstract | Summarise the research question, population, design, methods including intervention/s (independent variable/s) and target behaviour/s and any other outcome/s (dependent variable/s), results, and conclusions | P.2, Abstract |
| INTRODUCTION | | |
| 3 Scientific background | Describe the scientific background to identify issue/s under analysis, current scientific knowledge, and gaps in that knowledge base | PP. 3-4, Background |
| 4 Aims | State the purpose/aims of the study, research question/s, and, if applicable, hypotheses | P. 4, Objectives |
| METHODS | | |
| DESIGN | | |
| 5 Design | Identify the design (e.g., withdrawal/reversal, multiple-baseline, alternating-treatments, changing-criterion, some combination thereof, or adaptive design) and describe the phases and phase sequence (whether determined a priori or data-driven) and, if applicable, criteria for phase change | P. 5, Study Design |
| 6 Procedural changes | Describe any procedural changes that occurred during the course of the investigation after the start of the study | NA |
| 7 Replication | Describe any planned replication | NA |
| 8 Randomisation | State whether randomisation was used, and if so, describe the randomisation method and the elements of the study that were randomized | P. 5, Study Design |
| 9 Blinding | State whether blinding/masking was used, and if so, describe who was blinded/masked | P. 5, Study Design |
| PARTICIPANT/S or UNIT/S | | |
| 10 Selection criteria | State the inclusion and exclusion criteria, if applicable, and the method of recruitment | P. 5, Participants |
| 11 Participant characteristics | For each participant, describe the demographic characteristics and clinical (or other) features relevant to the research question, such that anonymity is ensured | P. 11, Results; Table 2 |
| CONTEXT | | |
| 12 Setting | Describe characteristics of the setting and location where the study was conducted | P. 5, Participants |
| APPROVALS | | |
| 13 Ethics | State whether ethics approval was obtained and indicate if and how informed consent and/or assent were obtained | P. 10, Ethical Considerations |
| MEASURES and MATERIALS | | |
| 14 Measures | Operationally define all target behaviours and outcome measures, describe reliability and validity, state how they were selected, and how and when they were measured | PP. 8-10, Measures; Figure 1 |
| 15 Equipment | Clearly describe any equipment and/or materials (e.g., technological aids, biofeedback, computer programs, intervention manuals or other material resources) used to measure target behaviour/s and other outcome/s or deliver the interventions | P. 5, Study Design |
| INTERVENTIONS | | |
| 16 Intervention | Describe intervention and control condition in each phase, including how and when they were actually administered, with as much detail as possible to facilitate attempts at replication | PP. 5-7, Study Design, CBT Exercises |
| 17 Procedural fidelity | Describe how procedural fidelity was evaluated in each phase | P. 12, Dropout and Dose |
| ANALYSIS | | |
| 18 Analysis | Describe and justify all methods used to analyse data | P. 10, Statistical Analyses |
| RESULTS | | |
| 19 Sequence completed | For each participant, report the sequence actually completed, including the number of trials for each session for each case. For participant/s who did not complete, state when they stopped and the reasons | P. 12, Dropout and Dose (means and range of compliance rates) |
| 20 Outcomes and estimation | For each participant, report results, including raw data, for each target behaviour and other outcome/s | PP. 12-14, Intervention Effect, Table 2, Figure 2 |
| 21 Adverse events | State whether or not any adverse events occurred for any participant and the phase in which they occurred | P. 12, Dropout and Dose |
| Discussion | | |
| 22 Interpretation | Summarise findings and interpret the results in the context of current evidence | PP. 14-15 |
| 23 Limitations | Discuss limitations, addressing sources of potential bias and imprecision | P. 16, Limitations |
| 24 Applicability | Discuss applicability and implications of the study findings | PP. 15-16, Efficacy, Conclusions |
| DOCUMENTATION | | |
| 25 Protocol | If available, state where a study protocol can be accessed | P. 10, Ethical Considerations |
| 26 Funding | Identify source/s of funding and other support; describe the role of funders | P. 17, Funding |
